# New Roadmaps for Non-muscle-invasive Bladder Cancer With Unfavorable Prognosis

**DOI:** 10.3389/fchem.2020.00600

**Published:** 2020-07-31

**Authors:** Katia Pane, Peppino Mirabelli, Luigi Coppola, Ester Illiano, Marco Salvatore, Monica Franzese

**Affiliations:** ^1^IRCCS SDN, Naples, Italy; ^2^Andrological and Urogynecological Clinic, Santa Maria Terni Hospital, University of Perugia, Terni, Italy

**Keywords:** bladder cancer, *Bacillus Calmette-Guérin*, omics, biomarker, prognostic factor, microbiome, immunotherapy, non-muscle-invasive bladder cancer

## Abstract

About 70% of bladder cancers (BCs) are diagnosed as non-muscle-invasive BCs (NMIBCs), while the remaining are muscle-invasive BCs (MIBCs). The European Association of Urology (EAU) guidelines stratify NMIBCs into low, intermediate, and high risk for treatment options. Low-risk NMIBCs undergo only the transurethral resection of the bladder (TURB), whereas for intermediate-risk and high-risk NMIBCs, the transurethral resection of the bladder (TURB) with or without *Bacillus Calmette-Guérin* (BCG) immune or chemotherapy is the standard treatment. A minority of NMIBCs show unfavorable prognosis. High-risk NMIBCs have a high rate of disease recurrence and/or progression to muscle-invasive tumor and BCG treatment failure. The heterogeneous nature of NMIBCs poses challenges for clinical decision-making. In 2020, the EAU made some changes to NMIBCs BCG failure definitions and treatment options, highlighting the need for reliable molecular markers for improving the predictive accuracy of currently available risk tables. Nowadays, next-generation sequencing (NGS) has revolutionized the study of cancer biology, providing diagnostic, prognostic, and therapy response biomarkers in support of precision medicine. Integration of NGS with other cutting-edge technologies might help to decipher also bladder tumor surrounding aspects such as immune system, stromal component, microbiome, and urobiome; altogether, this might impact the clinical outcomes of NMBICs especially in the BCG responsiveness. This review focuses on NMIBCs with unfavorable prognoses, providing molecular prognostic factors from tumor immune and stromal cells, and the perspective of urobiome and microbiome profiling on therapy response. We provide information on the cornerstone of immunotherapy and new promising bladder-preserving treatments and ongoing clinical trials for BCG–unresponsive NMIBCs.

## Introduction

In the United States, urothelial carcinoma of the bladder represents the most frequent urothelial neoplasm, and the fourth most common cancer in men (McConkey and Lerner, 2019; Siegel et al., 2020). Approximatively 70% of bladder cancer (BC) are non-muscle-invasive (NMIBCs), also known as “superficial” cancer, whereas advanced stages are muscle-invasive BCs (MIBCs) or metastatic BCs (Kamat et al., 2016). According to the European Association of Urology (EAU) guidelines, NMIBCs are distinct into low, intermediate, and high risk (Babjuk et al., 2019, 2020). The standard of care recommends for low-risk NMIBCs only the transurethral resection of the bladder (TURB); for intermediate- and high-risk NMIBCs, standard of care recommends the transurethral resection of the bladder (TURB) with or without *Bacillus Calmette-Guérin* (BCG) immune or chemotherapy (Babjuk et al., 2019). On the contrary, MIBCs undergo radical cystectomy, radiotherapy, and/or chemotherapy even alone (Babjuk et al., 2019; McConkey and Lerner, 2019). Genitourinary cancers are the most likely responsive to immunotherapy (Lalani and Sonpavde, 2019); however, about 20–30% of BCs have unfavorable to very unfavorable prognoses (Babjuk et al., 2019). High-risk NMIBCs show a greater propensity for disease recurrence and/or progression to muscle-invasive tumor, even after optimal BCG immunotherapy (Tse et al., 2019). NMIBCs require a better risk stratification due to clinical and molecular heterogeneity also in BCG responsiveness, which poses a major challenge for clinical decision-making. In 2020, the EAU made changes for NMIBC BCG failure definitions and treatment options (Table 1), since novel promising bladder-preserving treatments are currently under evaluation. Moreover, it has highlighted the need for reliable molecular markers for improving the predictive accuracy of currently available risk tables (Babjuk et al., 2019; Soukup et al., 2020).

**Table 1:** European Association of Urology (EAU) 2020 guidelines for the treatment of *Bacillus Calmette-Guérin* (BCG) failure (Table 7.7 EAU guidelines).

**Category**	**Treatment options**	**Strength rating**
BCG-unresponsive	1. Radical cystectomy (RC)	Strong
	2. Enrollment in clinical trials assessing new treatment strategies	Weak
	3. Bladder-preserving strategies in patients unsuitable or refusing RC	Weak
Late BCG relapsing: T1Ta/HG recurrence >6 months or CIS >12 months of last BCG exposure	1. Radical cystectomy or repeat BCG course according to individual situation	Strong
	2. Bladder-preserving strategies	Weak
LG recurrence after BCG for primary	1. Repeat BCG or intravesical chemotherapy	Weak
	2. Radical cystectomy	Weak

In the last decades, the increasing number of biobanks infrastructures (Coppola et al., 2019) has allowed the prompt availability of quality-controlled biological samples to be processed using next-generation sequencing (NGS) technologies (Chakraborty et al., 2018). The advent of NGS technologies and radiomics has revolutionized the approach to study disease biology, augmenting precision oncology (Incoronato et al., 2017; Zanfardino et al., 2019a,b; Castaldo et al., 2020). In BC research, the integration of NGS technologies with cutting-edge approaches might help to decipher also other tumor-relevant aspects such as immune and stromal component, microbiome, and urobiome modifications. This might impact the clinical outcomes of NMIBCs, especially for the BCG responsiveness.

This review focuses on NMIBCs with unfavorable prognosis providing interesting molecular prognostic factors from tumor closely related immune and stroma cells and the perspective of urobiome and microbiome profiling on therapy response. We illustrate the cornerstone of immunotherapy and new promising bladder-preserving treatments for BCG-unresponsive including the ongoing Food and Drug Administration (FDA)-approved clinical trials.

## Need For Reliable Molecular Bladder Cancer Biomarkers and Cutting-Edge Approaches

Currently, NMIBC risk stratification is based especially on clinical–pathological parameters such as grade (Babjuk et al., 2019). As opposed to other cancers such as prostate, BCs lack prognostic molecular markers used in clinical practice. NMIBC new prognostic factors are coming from tumor–host biology studies (Cooley et al., 2020). Indeed, innate and adaptive immune cells as well as stromal components surrounding tumor may have a prognostic value especially for evaluating BCG responsiveness in NMIBCs. Recently, Mezheyeuski et al. (2020) analyzed five cancer-associated fibroblast (CAF) markers, stroma-based, alpha smooth muscle actin (ASMA), CD90/Thy-1, fibroblast activation protein (FAP), platelet-derived growth factor receptor-alpha and -beta (PDGFRa-b) with survival and histopathological characteristics in 344 BC patients (231 NMIBCs, 113 MIBCs). Cluster analysis of stromal marker-based patient stratification identified a FAP-dominant patient cluster as an independent marker for shorter 5-year survival [Hazard Ratio, HR (95% confidence interval) 2.25 (1.08–4.67), *p* = 0.030]. Other studies on immunomodulatory properties rely on CD8a and revealed a potential minority of cases with CD90-defined stroma and high CD8a T cell infiltration showing a good prognosis of more than 80% 5-year survival (Mezheyeuski et al., 2020). Chu et al. (2020) described an innovative approach using indoleamine 2,3-dioxgenase 1 (IDO1) inhibitors in combination with immunotherapies in MIBCs and NMIBCs; the use of specific molecules such as indoximod, epacadostat, and linrodostat permit the immune microenvironment manipulation and increase of sensitivity to existing therapies with the goal of preventing the immune escape of cancer (Chu et al., 2020). In a retrospective study of high-grade pT1NMIBCs after TURB, the abundance of stromal tumor-infiltrating lymphocytes (TILs) associated with tumor invasion depth (Rouanne et al., 2019). Guillamón et al. (2019) isolated natural killer cells (NK) from BC peripheral blood for classifying patient risk which may be combined with BC histopathology. The mutation rate of genetic alterations may have an important prognostic value. Telomerase reverse transcriptase gene promoter (TERTp) mutations represent a frequent genetic event in BC. Batista et al. (2020) screened 125 NMIBC high-risk patients treated with BCG therapy (referred as BCG-NMIBC) for TERTp mutations, TERT rs2853669 single-nucleotide polymorphism, and fibroblast growth factor receptor 3 (FGFR3) hot spot mutations. TERTp mutations were found in 56% of BCG-NMIBC and were not associated with tumor stage or grade. FGFR3 mutations were found in 44.9% of the cases and were not associated with tumor stage or grade nor with TERTp mutations. The TERT rs2853669 single-nucleotide polymorphism was associated with tumors of a higher grade. The specific c.1-146 G > A TERTp mutation was an independent predictor of non-recurrence after BCG therapy (Hazard Ratio, HR 0.382; 95% confidence interval—0.150–0.971, *p* = 0.048) (Batista et al., 2020). In clinical practice, to date, we are still far from reliable molecular prognostic biomarkers due especially to heterogeneous results.

## Perspectives of Bladder Microbiome and Urobiome: a New Opportunity for a Predictive Response?

NGS technologies enable the study of the microbiome (Marchesi et al., 2016; Yoshida et al., 2018; Forkosh and Ilan, 2019). Microbial dysbiosis impacts several human diseases including bladder carcinogenesis (Bajic et al., 2019b). However, the mechanistic links between microbiome signatures and bladder pathogenesis are still unknown. Urobiome studies may open the way to understand how bladder microbiota affects immunotherapy response leading to BCG-unresponsive patients (Wolfe and Brubaker, 2019).

Urinary microbiome through the 16S ribosomal RNA sequencing and expanded quantitative urine culture (EQUC) enable unculturable and/or rare microbes detection (Ferreira et al., 2010; Karstens et al., 2018; Govender et al., 2019). The core step in microbiome analysis is the taxonomic classification of the representative sequences and clustering of operational taxonomic units (OTUs). To assess the association between microbiome signatures and clinical phenotype, useful bioinformatic tools are PERMANOVA-S (Tang et al., 2016) and MiRKAT (Zhao et al., 2015). Bladder microbiome analysis showed that the most abundant genera are *Lactobacillus* (15%), followed by *Corynebacterium* (14.2%), *Streptococcus* (11.9%), *Actinomyces* (6.9%), and *Staphylococcus* (6.9%) (Bersanelli et al., 2019). Innovative drug strategies based on precise antimicrobial peptides might boost the host immune system to ensure bladder microbiome homeostasis (de la Fuente-Nunez et al., 2017; Gaglione et al., 2019). Novel urinary biomarkers proved to be endowed with prognostic and diagnostic value in urological malignancies derived from exosomes, such as H2B1K and alpha-1 antitrypsin (Wu et al., 2019).

Recent studies evaluated microbiome composition using 16S ribosomal RNA sequencing of urine specimens from healthy individuals vs. urothelial carcinoma patients with heterogeneous results especially because both studies used a small number of patients (Xu et al., 2014; Bučević Popović et al., 2018). Other studies have clearly shown the increase in bacterial species in BC patients compared to control patients, correlating them with the high risk of disease progression and indicating them as possible new biomarkers to further stratify patients. Although exciting, urinary microbiome signature needs precautions due to several issues associated with sample collections and management of the biological sample, sex factors such as age, menopausal status, sex steroid hormones, and body mass index (Karstens et al., 2018; Bajic et al., 2019b).

## Modern Anticancer Therapy

**Figure 1:**
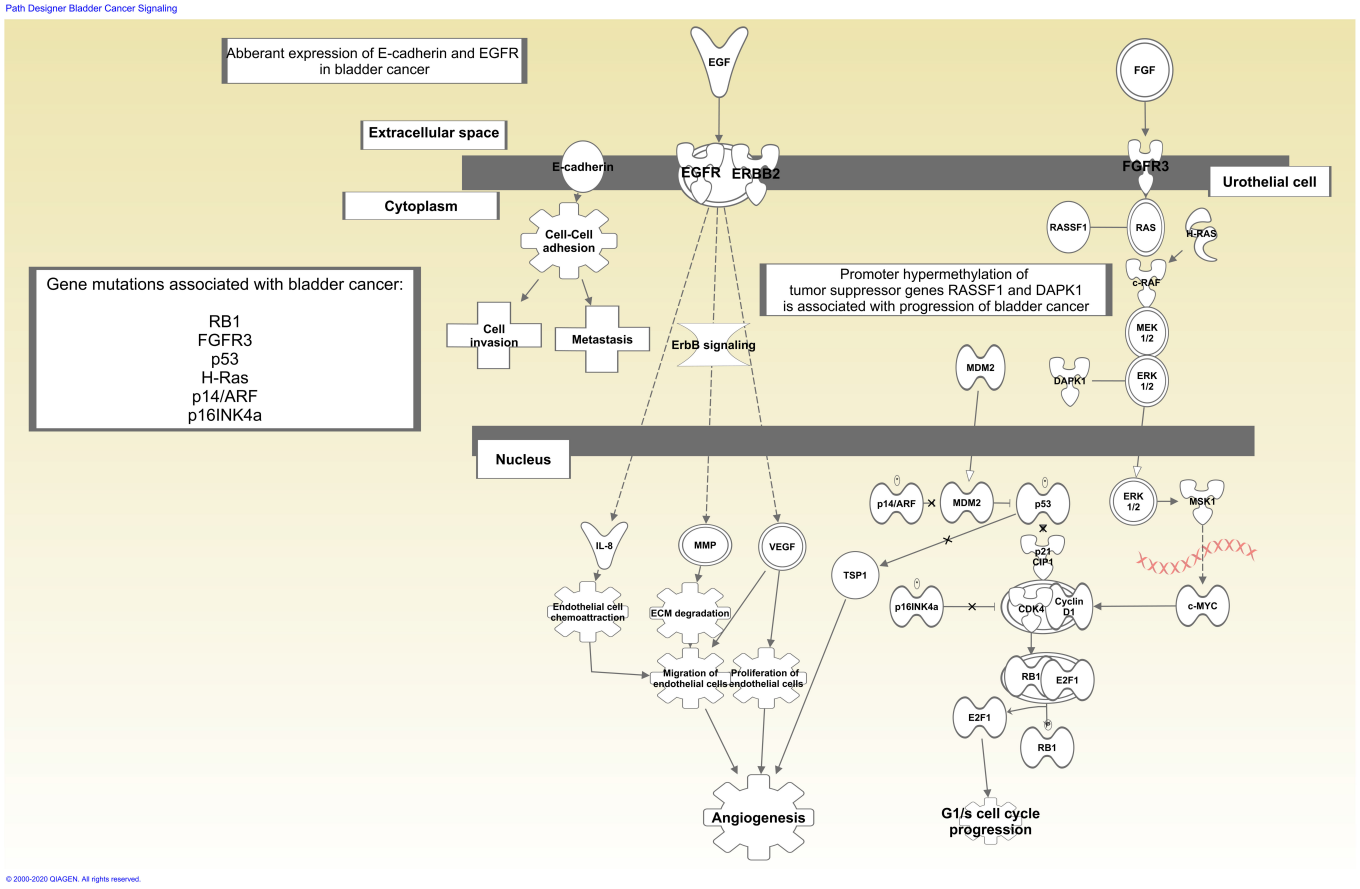
Schematic representation of bladder cancer signaling. Drug therapy based on immunotherapy affects tumor biology enhancing host immune response. In contrast, single arm or multi-target drugs may affect one single target or more targets within the same or multiple biological pathways underlying bladder cancer. Signaling generated through the use of IPA (QIAGEN Inc., https://www.qiagenbioinformatics.com/products/ingenuity-pathway-analysis).

A panel of potential drug targets underlying BC signaling (Krämer et al., 2014) is schematically represented in Figure 1. In recent years, there has been a growing demand for BCG-unresponsive salvage treatments as alternative to radical cystectomy. Moreover, immunotherapies and new therapeutic options, listed in Table 2, may be administered as single arm (monotherapy) but also as a combination and even multitarget drugs could be available in the near future (see below).

**Table 2:** Modern therapeutic approaches for bladder cancer based on intravesical, intravenous, or subcutaneous applications and some clinical trials completed or with ongoing recruitment.

**Indication**	**Clinical trial^a^^,^^b^**	**Agent**	**Therapy approach/administration**
Metastatic bladder cancer	FDA approved	Atezolizumab (IgG1 anti-PD1 antibody)	Monotherapy/intravenous
Metastatic bladder cancer	FDA approved	Nivolumab (IgG4 anti-PD1 antibody)	Monotherapy/intravenous
Metastatic bladder cancer	FDA approved	Pembrolizumab (IgG4 anti-PD1 antibody)	Monotherapy/intravenous
Metastatic bladder cancer	FDA approved	Avelumab (IgG1 anti-PD1 antibody)	Monotherapy/intravenous
Metastatic bladder cancer	FDA approved	Durvalumab (FcR anti-PD1 antibody)	Monotherapy/intravenous
Bladder cancer	PRIME NCT02326168^a^ (Early Phase 1)	BCG vaccine	Monotherapy/percutaneous BCG vaccine
Delivery system for several neoplasms including stage III and stage IV bladder cancer	NCT02646319^a^ (Early Phase 1)	Nanoparticle albumin-bound rapamycin in treating patients with advanced cancer and mTor mutations	Monotherapy/intravenous
Intermediate-risk recurrent NMIBC	NCT03167151^b^ (August 2021)	Pembrolizumab (IgG4 anti-PD1 antibody)	Monotherapy/intravesical pembrolizumab or intravenous pembrolizumab
Bladder cancer	NCT04162704^b^ (October 2022)	Device: EpiCheck	Monotherapy/evaluation of predictor tumor response after intravesical mitomycin
Recurrent NMIBC patients and prior history of low- or intermediate-risk	NCT03914794^b^ (May 2023)	Pemigatinib (FGFR inhibitor)	Monotherapy/oral
High-risk NMIBC BCG–unresponsive	NCT02625961^b^ (July 2023)	Pembrolizumab (IgG4 anti-PD1 antibody)	Monotherapy/intravenous
NMIBC BCG-refractory	NCT03759496^b^ (December 2021)	Durvalumab (FcR anti-PD1 antibody)	Monotherapy/intravesical
NMIBC low-risk	NCT03081858^b^ (May 2022)	Proliposomal intravesical paclitaxel formulation (PLIP)	Monotherapy/intravesical
NMIBC tumor progression	NCT01373294^a^ (Phase 2)	BCG plus lenalidomide	Combination therapy/BCG intravesical and oral lenalidomide
BCG-refractory high-risk NMIBC patients who are medically unfit for/refuse cystectomy	NCT03053635^a^ (Phase 1)	TLD1433 infusion and photodynamic therapy (PDT)	Combination therapy/Intravesical photodynamic therapy (PDT) and TLD1433
Superficial bladder cancer patients	NCT00004122^a^ (Phase 2)	BCG plus interferon alpha 2b	Combination therapy/BCG and Interferon alfa 2b intravesical
Recurrent bladder tumor and >stage II	NCT01938573^a^ (Phase 1/Phase 2)	Combination of sirolimus, cisplatin, and gemcitabine hydrochloride	Combination therapy/intravenous
Advanced or metastatic bladder cancer	NCT01828736^a^ (Phase 2)	Combination of biological and chemotherapy: Trastuzumab Gemcitabine Carboplatin Cisplatin	Combination therapy/intravenous
NMIBC BCG-unresponsive	NCT03945162^b^ (May 2022)	TLD-1433 bladder infusion and photodynamic therapy (PDT)	Combination therapy/intravesical photodynamic therapy with TLD-1433
NMIBC	NCT04179162^b^ (November 2022)	BCG and gemcitabine	Combination therapy/intravesical
High-risk NMIBC FGFR mutations or fusions	NCT04172675^b^ (May 2026)	Erdafitinib plus gemcitabine or mitomycin C	Combination therapy/oral
High-risk NMIBC	NCT03711032^b^ (November 2024)	Pembrolizumab plus BCG	Combination therapy/BCG intravesical therapy and pembrolizumab intravenous
High-risk NMIBC	NCT04149574^b^ (August 2030)	Nivolumab plus BCG	Combination therapy/BCG and nivolumab intravesical
Known or suspected NMIBC	NCT02660645^b^ (December 2020)	Drug and device: blue light cystoscopy with hexaminolevulinate hydrochloride and Karl Storz D-light C photodynamic diagnostic (PDD) system	Combination therapy/instillation of hexaminolevulinate hydrochloride in bladder prior to cystoscopy
NMIBC BCG-naive	POTOMAC NCT03528694^b^ (November 2024)	Durvalumab (MEDI4736) plus BCG	Combination therapy/intravenous drug and BCG intravesical therapy
High-risk NMIBC and MIBC	NCT03636256^b^ (November 2020)	NanoDoce	Combination therapy/NanoDoce as a direct injection to the bladder wall immediately after TURB and as an intravesical instillation.
High-risk NMIBC BCG-unresponsive	NCT03022825^b^ (January 2023)	IL-15 superagonist complex ALT-803 and BCG	Combination therapy/BCG and ALT-803 intravesical
Advanced bladder cancer BCG recurrent or metastatic bladder cancer	NCT00003167^a^ (Phase 1)	Adenovirus p53 gene therapy (Ad5-p53)	Gene therapy/intravesical
NMIBC BCG failure	NCT02365818^a^ (Phase 2)	CG0070, oncolytic virus expression GMCSF	Gene therapy/intravesical
Transitional cell cancer of the bladder	NCT00070070^a^ (Phase 1)	NY-ESO-1 peptide vaccine mixed with BCG	Vaccine therapy/intradermally
NMIBC	NCT03421236^b^ (March 2021)	Ty21a bacteria vaccine	Vaccine therapy/intravesical

*Source: https://clinicaltrials.gov/ accessed on June 2020*.

a *We included some clinical trials from no. 423 studies for whom recruiting is completed with clinical trial phase status*.

b *We included some clinical trials from no. 21 studies for whom recruiting is ongoing with estimated study completion date*.

### Immunotherapy of Bladder Cancer: New Frontiers

During the 1990s, the immune checkpoint key proteins, cytotoxic T lymphocyte-associated protein 4 (CTLA-4), programmed cell death protein (PD-1), and programmed death-ligand 1 (PD-L1), revolutionized cancer therapy (Korman et al., 2006; Okazaki and Honjo, 2007). Currently, immunotherapy, together with radiotherapy and chemotherapy, represents standard clinical care for cancer including BC (Butt and Malik, 2018). Immunotherapeutic drugs inhibit immune checkpoint pathways, enabling the immune system to fend off cancer cells through cytotoxic T killer cells (Korman et al., 2006). BC can cause inflammation by attracting inflammatory cells to the cancer cell site which led to the activation of different components of the immune system such as tumor-associated macrophages (TAMs), neutrophils, and granulocytes. Nevertheless, a crucial role is played by T lymphocytes; this immune system cell component has an important role in tumor development and progression; regulatory T cells have been shown to be involved in maintaining self-tolerance modulating the antitumor immune response and therefore potential cancer growth and spread. These evidences have highlighted that lymphocytes are intrinsically linked to tumorigenesis in the bladder, acting as target for BC immunomodulatory therapy. To date, five FDA-approved immunotherapy agents are commonly used for metastatic BC treatment (Table 2). Nivolumab and pembrolizumab are anti-PD1 drugs, tremilimumab and ipilimumab are anti-CTLA-4 drugs; furthermore, MBG453 is a TIM3 inhibitor. Through the inhibition of PD-L1, the tumor cells are targeted; for this approach, the molecules used are durvalumab, avelumab, and atezolizumab. Further strategies include a modulation of regulatory T cells by targeting CD25 (daclizumab) or alternatively CCR4 (mogamulizumab). Genitourinary cancers are most likely responsive to immune checkpoint inhibitors such as PD-1 and PD-L1 antibodies (Lalani and Sonpavde, 2019). The promising POTOMAC study compares the effect of combining durvalumab plus BCG vs. BCG alone both in high-risk NMIBC patients; the BMS-986205 study compares the administration of nivolumab alone or in combination with BCG in BCG-unresponsive patients. The goal of both studies has been to evaluate the safety and effectiveness and a possible decrease of costs for patient management compared to standard therapy. As shown in Table 2, several clinical trials are designed to investigate different therapeutic approaches in BC. Combination therapies, based on BCG immunotherapy and different chemical (NCT01240824) or biological compounds (NCT00004122) or vaccines (NCT00070070), or combination therapies encompass also combination chemotherapies (Steinberg et al., 2018) especially in recurrent and advanced BC (NCT01938573 and NCT01828736, respectively). In addition, Table 2 shows ongoing clinical trials that might have clinical implications on the use of these drugs in the near future. Many BC trials are exploring immunotherapy, vaccines, chemotherapy, or gene therapy efficacy for BCG-unresponsive disease, advanced, recurrent, and metastatic BC. For a comprehensive list of drugs for several BC phenotypes, see Butt and Malik (2018), Rouanne et al. (2018), and Soria et al. (2019), while for specific NMIBCs, BCG-unresponsive, see Tse et al. (2019).

### *Bacillus Calmette-Guérin* Intravesical Therapy

According to the EAU guidelines, the gold standard immunotherapy for intermediate- and high-risk NMIBC is intravesical full-dose BCG instillation (instillations 3, 6, 12, 18, 24, 30, and 36 months), for 1 year (intermediate-risk) or for 1–3 years (high-risk), respectively (Butt and Malik, 2018; Babjuk et al., 2019; Bajic et al., 2019a). In highest-risk tumors, e.g., associated with concurrent bladder carcinoma *in situ* (CIS) and BCG failure, radical cystectomy is recommended (Babjuk et al., 2019).

BCG immunotherapy ensures NMIBC treatment also in the elderly, not eligible for cisplatin systemic chemotherapy (Soria et al., 2019). Although BCG mode of action is still not completely understood, its efficacy in superficial bladder carcinoma might be achieved through the local enhancement of the immune response, recruitment of inflammatory cells, and release of cytokines (Lawrence et al., 2013; Song et al., 2019). Moreover, BCG therapy has been shown to reduce tumor progression and recurrence rate in NMIBC treated patients compared with NMIBC who underwent TURB alone or TURB plus chemotherapy (Lawrence et al., 2013; Song et al., 2019).

A debated point which requires intense further investigation is to understand if BCG substrains, generated over time, could alter the host response. The main substrains used are Russia, Moreau, Japan, Sweden, and Birkhaug (elimination of region of differentiation 1) and BCG Prague, Glaxo, Danish, Tice, Frappier, and others (deletion region of differentiation 2) (Hayashi et al., 2010; Kasempimolporn et al., 2018). However, findings available so far are not sufficient to support more effectiveness of specific BCG strain over another.

### *Bacillus Calmette-Guérin* Unresponsive Patients: New Treatments

According to established EAU guidelines, in NMIBC intermediate-risk, 1-year full-dose intravesical BCG treatment or full-dose intravesical BCG for 1–3 years for high-risk NMIBC is recommended; however, a subset of NMIBC patients do not respond to BCG treatment (BCG failure) and may recur or progress with the neoplasm (Packiam et al., 2019). In BCG-unresponsive patients, poor therapeutic alternatives are available so far; radical cystectomy is the standard of care for the majority of BCG-refractory; intravesical valrubicin remains the only agent that is FDA approved in BCG-refractory patients with CIS (Cookson et al., 2014; Babjuk et al., 2019). Nevertheless, NMIBCs include disease entities with distinct prognoses. In 2020, EAU made some changes to the guidelines on NMIBC BCG failure treatments. The EAU recommends, in addition to radical cystectomy, bladder-preserving strategies with new treatment options or enrollment in clinical trials, although the strength rating for this latter chance is weak. Table 2 shows some clinical trials designed for BCG-refractory high-risk NMIBCs such as NCT03053635 and NCT00003167.

Innovative strategies deal with the improvement of BCG efficacy through recombinant BCG and priming–boosting strategy or the use of alternative systems such as (i) viruses, (ii) bacteria, and (iii) chemotherapeutic drugs. In regard to the therapeutic enhancement of the BCG immunotherapy, initial stimulation by subcutaneous BCG vaccination allowed to develop a more effective immune response following intravesical BCG bladder instillation (Svatek et al., 2018). Furthermore, biotechnologically advanced strategies have allowed the intravesical BCG to acquire characteristics such as to develop a beneficial immune response through the expression of specific bacterial antigens or through the modification of the genetic patrimony for the expression of molecules (cytokines or chemokines) with immunomodulatory properties (Begnini et al., 2015; Burggraaf et al., 2019).

Innovative strategies other than BCG therapy involve:

- *Viruses* can be delivered into the bladder through the current procedures and can have higher effectivity than BCG; by targeting specifically cancer cells, viruses could reduce adverse events compared with the use of the bacillus (Taguchi et al., 2017). Several ongoing clinical trials are testing this promising new strategy (Tse et al., 2019).
- *Bacteria* utilization could be another possible strategy in the unresponsive NMIBC or in NMIBC patients who have developed side effects. The use of other Mycobacteria substrains is being studied for BC treatment. *Mycobacterium phlei* represents an alternative in the treatment of unresponsive BCG patients; in addition, in preclinical studies, *Mycobacterium brumae* was evaluated as a safe and efficacious candidate for NMIBC (Morales and Cohen, 2016; Noguera-Ortega et al., 2016)
- *Intravesical chemotherapeutic drugs*, alone or in combination, are used for high-risk NMIBC such as pirarubicin, gemcitabine, and epirubicin (Kang et al., 2016); intravesical application of mitomycin C is a chemotherapeutic agent most used in patients who do not respond to BCG (Fankhauser et al., 2020). The latter approach is also useful for patients who have developed significant side effects; the combination of BCG treatment with chemotherapeutic agents has demonstrated the reduction of side effects and an improvement of tolerability to BCG (Huang et al., 2019).
- *Cancer vaccines*, novel biological drugs such as BCG vaccines have been evaluated to elicit an immune response and modulate side effects using recombinant BCG strains able to stimulate Th1 immune cells as well as induce cytokine release (Cho et al., 2019; Rodriguez et al., 2019).

### Monotherapy, Combination Therapy, and Multi-Target Therapy

Combination therapies based on immunomodulators such as checkpoint inhibitors have shown a synergistic effect to augment the immune response (Marshall and Djamgoz, 2018).

A discrete amount of studies are based on combination therapy with chemotherapeutic drugs, intravesical BCG, and immune checkpoint inhibitors, as some trials reported in Table 1 (Aggen and Drake, 2017). Currently, an FDA-approved combination therapy is based on intravesical gemcitabine and cisplatin for NMIBCs (Rayn et al., 2018).

As shown in Table 2, several clinical trials are designed to investigate combination therapies based on BCG immunotherapy and different chemical (NCT01240824) or biological compounds (NCT00004122) or vaccines (NCT00070070). Combination therapies encompass also combination chemotherapies (Steinberg et al., 2018) especially in recurrent and advanced BC including (NCT01938573 and NCT01828736, respectively). Furthermore, photodynamic immunotherapy emerged recently to stimulate the immune response in NMIBC BCG-refractory or intolerant to BCG treatment as well (NCT03053635) (Lee et al., 2013).

One frontier of medicinal chemistry is polypharmacology (Proschak et al., 2019). Benedetti et al. (2015) reviewed the immuno-oncological dynamic interactions to design multi-target modulators.

A multitarget drug can be considered as a key drug that opens multiple locks, able to inhibit multiple molecules within cascade signaling or within crosstalk pathways (de Oliveira Viana et al., 2018). Among multitarget modulators, epi-enzymes, histone deacetylases (HDACs), and DNA methyltransferase (DNMT) families represent the most studied drug targets for several cancer types (Benedetti et al., 2015; Lu et al., 2018). One promising compound achieved by this design approach is levosimendan, which proved efficacy on several cancer cell lines including BC urothelial carcinoma (Lim et al., 2019).

## Concluding Remarks

As opposed to other tumors, to date, there is not a real “routine” tumor prognostic molecular marker in BC clinical practice (Koncina et al., 2020). At diagnosis, most BCs are non-muscle-invasive and can be curable according to risk stratification into low, intermediate, and high risk; however, the minority of patients who do not respond to BCG immunotherapy poses a great challenge for the clinical decision-making. How to predict in advance patients with intermediate- or high-risk NMIBC, who will respond to BCG immunotherapy, or who will progress or recur into muscle-invasive phenotype is an open question. Furthermore, recurrence after BCG therapy for the primary tumor is still a challenge in the management of BC.

A better assessment of the NMIBC risk stratification and prognosis will provide significant medical, economic, and societal benefits. To overcome the high disease recurrence rate of NMIBCs and BCG failure, novel NGS techniques explore the association between bladder microbiome and therapy response and could provide BCG response biomarker in advance. To date, the possibility of using urobiome signatures as non-invasive biomarkers is still unlikely especially because contamination from other body districts is very high. For this reason, clinical studies focusing on patients catheterized urine from surveillance cystoscopy, rather than voided urine, are largely encouraged. Microbiome composition may be helpful to understand why some patients with NMIBC after BCG therapy have disease recurrences or progressions and others remain cured over time. New treatment options for bladder-preserving strategies are under evaluation to improve therapy efficacy in support of precision medicine also for bladder cancer. Urologists have to face the lack of prognosis accuracy for providing information on treatment options to intermediate- or high-risk NMIBCs patients. This has strong consequences also on patient counseling. In this concern, molecular nomograms for predicting prognosis and treatment response in NMIBCs will be very helpful.

Collectively, advances in multi-omic studies suggest that continuous efforts from wet-lab might provide shortly reliable molecular prognostic biomarkers.

## Author Contributions

KP conceived and wrote the draft. All authors revised the manuscript and approved the final version and contributed to the conception of this work.

## Conflict of Interest

The authors declare that the research was conducted in the absence of any commercial or financial relationships that could be construed as a potential conflict of interest.

## Abbreviations

ASMA: Alpha smooth muscle actin
BC: Bladder cancer
BCG: *Bacillus Calmette-Guérin*
CIS: Carcinoma *in situ*
CTLA-4: Cytotoxic T-lymphocyte associated protein 4
DNMT: DNA methyltransferase
EAU: European Association of Urology
EQUC: Expanded quantitative urine culture
FAP: Fibroblast activation protein alpha
FGFR3: Fibroblast growth factor receptor 3
HDACs: Histone deacetylases
IDO1: Indoleamine 2,3-dioxgenase 1
lncRNA: Long noncoding RNA
MIBCs: Muscle-invasive bladder cancers
NGS: Next-generation sequencing
NMIBCS: Non-muscle-invasive bladder cancers
OTUs: Operational taxonomic units
PD-1: Programmed cell death protein
PDGFRa-b: Platelet-derived growth factor receptor alpha and beta
PD-L1: Programmed death-ligand 1
TAMs: Tumor associated macrophages
TERTp: Telomerase reverse transcriptase gene promoter
TILs: Tumor infiltrating lymphocytes
TURB: Transurethral Resection of the Bladder.
